# Improving the Accuracy of the Evaluation Method for the Interfacial Shear Strength of Fiber-Reinforced Thermoplastic Polymers through the Short Beam Shear Test

**DOI:** 10.3390/polym16070883

**Published:** 2024-03-23

**Authors:** Quan Jiang, Tetsuo Takayama, Akihiro Nishioka

**Affiliations:** Graduate School of Organic Materials Science, Yamagata University, 4-3-16 Jonan, Yonezawa 992-8510, Japannishioka@yz.yamagata-u.ac.jp (A.N.)

**Keywords:** injection molding, interfacial shear strength, mechanical properties, short beam shear test, short fiber-reinforced thermoplastics

## Abstract

Short fiber-reinforced thermoplastic polymers (SFRTPs) are commonly used in various molding methods due to their high specific elasticity and strength. To evaluate the interfacial strength, several determination methods have been proposed, including the interfacial shear strength (IFSS). In previous research, an IFSS evaluation method based on the short beam shear method was proposed. However, this method is only applicable to micrometer-sized fibers with high stiffness levels that are not easily bent. When utilizing cellulose fiber, the interfacial shear strength (IFSS) results frequently exhibit significant deviations. To tackle this issue, we suggest an enhanced experimental technique that employs beam-shaped specimens with welding points based on the short beam shear test. Furthermore, we conducted a three-dimensional analysis of the original method to determine the fiber orientation angle and IFSS. The outcomes were compared with previously reported determinations. The IFSS achieved through the novel method proposed in this paper exhibits high precision and reliability, rendering it suitable for use with soft and flexible fibers.

## 1. Introduction

In recent years, environmental pollution has become an increasingly severe issue worldwide. To address this issue, countries are emphasizing the weight reduction of vehicles that predominantly use fossil fuels, such as automobiles and aircraft.

**Figure 1:**
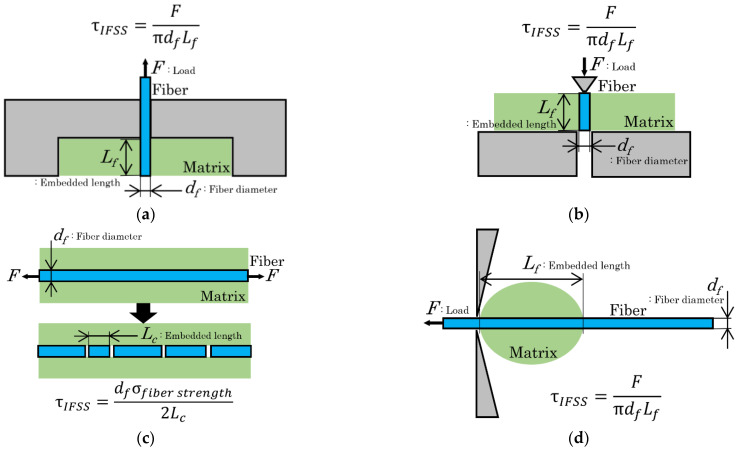
Method for ascertaining interfacial shear strength. (**a**) Pull-out method [2]. (**b**) Push-out method [3]. (**c**) Fragmentation method [5]. (**d**) Micro-droplet method [6].

One approach to weight reduction is to use short fiber-reinforced thermoplastics (SFRTPs), instead of metal, to make structural parts of thermoplastic composites. SFRTPs have higher specific strength levels and specific rigidity than metals. In this context, staple fibers are categorized as either inorganic or organic. Glass fiber [1] is a commonly used inorganic fiber due to its excellent properties, including high strength, flexibility, stiffness, and resistance to chemical damage. However, its recycling poses a challenge, necessitating the search for a fibrous reinforced phase that can replace glass fiber [2]. The United Nations prioritizes the use of environmentally friendly and recyclable composite materials in their SDG-related projects. Therefore, organic natural fibers have gained a significant level of attention. The development of new bio-composite materials is driven by several factors, including the lower cost of natural fibers (which are currently priced at one-third or less of the cost of glass fibers), weight reduction (as these fibers are half the weight of glass fibers), and the ease of recycling (natural fiber composites are easier to recycle). Furthermore, there is an increasing demand for environmentally friendly products [3]. In addition, low-cost manufacturing processes, such as injection molding, can be used to produce these components. The advantages of the injection molding method are its fast production speed, high efficiency, automation of operation, and a wide range of applicable resins. Even fiber-reinforced composite materials can achieve complex shapes and arbitrary size molding processes, and the products are precise in their size and easy to update. It can be used for mass production and complex shape products. Injection molding is suitable for molding processing fields such as mass production and complex shape products [4]. It is predicted that the use of natural fibers in composite materials will continue to expand. However, it should be noted that the tensile strength of natural fibers is lower than that of glass fibers [5,6,7]. Therefore, it is crucial to improve the mechanical properties of natural fiber composites. Various factors influence the mechanical properties of SFRTPs, such as tensile strength and impact strength. These factors can be categorized as originating from materials or the fiber–matrix interface. To evaluate the interfacial strength, interfacial shear strength (IFSS) is considered a crucial factor [8]. Various methods have been proposed for IFSS assessment, such as pull-out [9], push-out [10], fragmentation [11,12], and micro-droplet techniques [13]. Evaluation methods for fiber and matrix interfaces often use specialized samples, as shown in Figure 1. Pull-out, fragmentation, and micro-droplet methods are used to examine a single fiber and matrix test piece. These models provide clear advantages and can directly evaluate the interface. It is important to note that the test pieces required for these evaluation methods are typically at the micron-level.

Yunlong Li et al. reported the novel prediction methodology based on the interfacial stress impedance effect of magnetic fibers (MFs) for the non-destructive and real-time evaluation of temperature-dependent IFSS [14]. Through the impedance measurement and corresponding IFSS micromechanical test, a correlation model between the IFSS of the composite and the impedance of MFs is established. The IFSS at elevated temperatures calculated by this model are consistent with the results of the micromechanical test by more than 95% and allows for evaluating the IFSS of composites beyond the glass transition point. However, it should be noted that this testing method can only be limited to specific fiber structures and is not applicable to organic natural fibers. Wang et al. reported that the IFSS was found to have an inverse relationship with the test temperature [15], and they also found that the angle tilt has an impact on the interfacial strength test results. However, the deviation range measured using a single fiber pull-out experiment is large, and the effect of fiber volume fractions on interfacial strength is not considered. Yamaguchi et al. reported the effects of three maleic anhydride-modified polypropylene (MAPP) specimens with different MA contents and crystallinities on the IFSSs of PP and carbon fibers (CFs), which were investigated and compared through microdroplet tests [16]. It is demonstrated that the chemical interactions and crystallinity changes at the interface can cause a significant IFSS enhancement effect. Yu et al. [17], Wang et al. [18], and Ramaswamy et al. [19] reported the effects of a carbon fiber surface’s composite interfacial property. These results indicate that there is a positive correlation between the IFSS and mechanical properties. However, the research on additives and fiber surface treatments has not achieved high-precision interfacial strength that can establish quantitative models.

Majhi et al. reported a higher IFSS level between the cellulose fiber and the matrix material, which is responsible for the higher strength of composites [20]. Their results showed that the debonding load increases linearly with respect to the fiber’s embedded length. This proves that the fiber volume fraction affects the evaluation results of IFSS, and previous experiments have had difficulty controlling the fiber volume fraction. Falkenreck et al. [21], Barrera-Fajardo et al. [22], and Liu et al. [23] reported the effects of a natural fiber surface treatment on the mechanical properties of composites. These mechanical properties include tensile strength, bending strength, and surface friction, and so on. From the above literature, we have acknowledged that whether using inorganic glass fibers with higher stiffness [24] or organic natural fibers with lower stiffness [13], the test only utilizes a single fiber. The evaluation results for interfacial shear strength have a relatively high standard deviation range. However, it is worth noting that the determination of IFSS for the same fiber and matrix composition can have a wide range of deviation, especially when using cellulose fibers, which result in large deviations in IFSS.

Although these results demonstrate a correlation between mechanical strength and interfacial shear strength, the quantitative relationship between various performance parameters and the mechanical properties of composite materials is still relatively rare. It is important to note that Kallel et al. reported a quantitative relationship between parameters such as tensile strength and interfacial shear strength, fiber size, fiber tensile strength, critical fiber length and matrix elasticity, etc. [25]. However, the IFSS evaluation results with large deviations also lead to low accuracy in predicting tensile strength. Thus, it is not feasible to create a quantitative model that can integrate the determination results, strength, impact resistance, and other mechanical properties of SFRTPs. It can be seen that an accurate IFSS evaluation method urgently needs to be clarified.

Based on previous research, we proposed the IFSS evaluation method using the short beam shear method [26]. This method induces interlaminar shear failure by intentionally narrowing the distance between the supporting points in a three-point bending test. It takes advantage of the fact that higher shear stress occurs near the neutral plane than in a normal three-point bending test. The specimen is a short beam that has been injection-molded with discontinuous short fibers dispersed throughout. Near the neutral plane, the fibers are strongly oriented perpendicular to the flow direction [27]. Due to this fiber orientation characteristic, a small shear stress in the direction that is oriented at an angle close to parallel to the loading direction can initiate interface slips. If this slip occurs, it is expected that the stiffness of the specimen will drop discontinuously during the test. Based on this theory, we propose a method for calculating IFSS by taking two rigid, discontinuous drop points. The evaluation results of IFSS using short beam shear testing, as reported by our previous research, can be used to determine the impact absorption model [28]. The quantitative model is based on the impact energy absorption model of short glass fiber-reinforced polyethylene (PP/GF). The models of fiber pullout and fiber interface debonding, both of which are ways of absorbing impact energy, have a direct quantitative relationship with the IFSS and interfacial strength. However, it is important to note that natural fibers may bend or even become wound in injection-molded products [29]. When using natural fibers, the bending of fibers in injection-molded products leads to the low applicability of this method. This model needs further discussion and improvement. Therefore, it is necessary to further improve the accuracy of interfacial measurements.

This study discusses the addition of glass fibers and cellulose fibers to polypropylene and polystyrene, respectively. We predict that adjusting for fiber orientation distribution of the injection-molded short beam may improve measurement accuracy by ensuring the fiber plane distribution at the welding point. The short beam shear test is applied to two types of injection molding products: beam specimens (Beam) and beam specimens with a welding point (Beam with Weld). The measurement accuracy of IFSS is improved by controlling the injection flow path and using the plane distribution of welding points. The propriety of this approach was verified by comparing the IFSS calculation results of Beam and Beam with Weld. Furthermore, the fiber orientation angle was compared to the X-ray CT analysis results of the injection-molded products, clarifying the characteristics of the fiber orientation angle.

## 2. Materials and Sample Preparation

### 2.1. Materials

Polypropylene (PP, Novatec MA1B; Japan Polypropylene Corp., Tokyo, Japan) and polystyrene (PS, Toyo Styrene GPPS G210C; Toyo Styrene Co., Ltd., Tokyo, Japan) were used as matrices. As fibers, glass fibers (GFs, ECS 03 T351; Nippon Electric Glass Co., Ltd., Otsu, Japan) that were surface-modified with amino groups and hardwood cellulose fibers (CLFs) were used.

### 2.2. Sample Preparation

**Figure 2:**
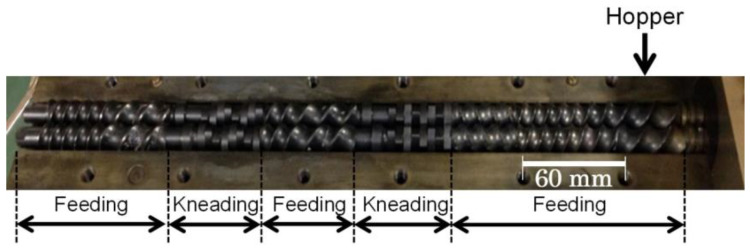
Screw configuration used for the twin-screw extruder.

The twin-screw extruder (IMC0-00; Imoto Machinery Co., Ltd., Kyoto, Japan) was used to add the materials. GFs were melt-mixed at a temperature of 230 °C and a screw speed of 60 rpm. CLFs were melt-mixed at a temperature of 200 °C and a screw speed of 60 rpm. The extruder had a 15 mm diameter screw, as shown in Figure 2, with a screw length-to-diameter ratio of 25. The mixing ratios are shown in Table 1. The GF content was fixed at 10wt%, and the CLF content was fixed at 12wt%. The 3 mm composite pellets were produced by pelletizing the melt-kneaded strands using a cold-cut pelletizer (Toyo Seiki Co., Ltd., Tokyo, Japan).

**Figure 3:**
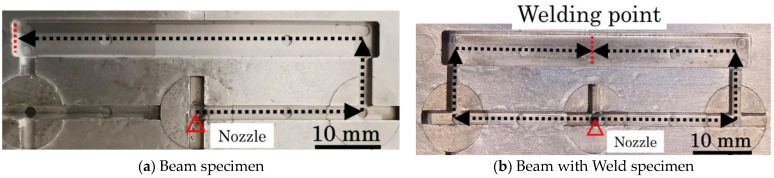
The mold cavity shapes and injection flow paths of the Beam and Beam with Weld specimens.

**Figure 4:**
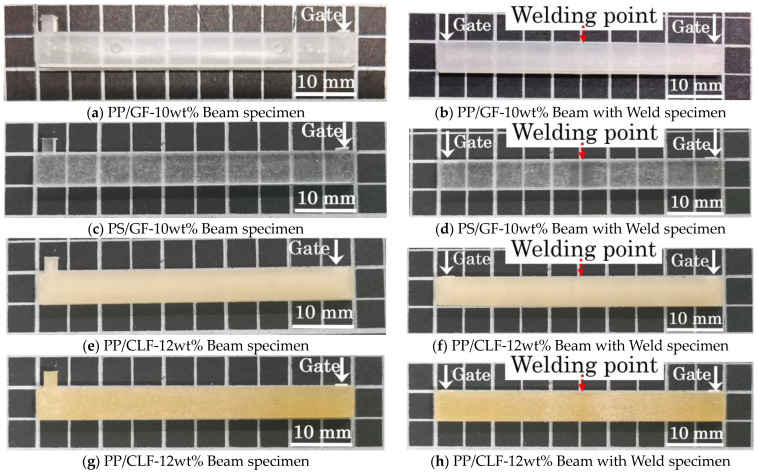
The geometries of the Beam and Beam with Weld specimens.

Two types of products were obtained by injection molding the composite material pellets using a micro-electric injection molding machine (C, Mobile0813; Shinko Sellbic Co., Ltd., Tokyo, Japan). Figure 3a displays the Beam specimens processed with a single flow direction. Figure 3b displays the Beam with Weld specimens that were processed by adjusting the flow path to form a weld in the center. The glass fiber (GF) content was adjusted to 10wt% by diluting the composite pellets with 10wt% fiber content composite pellets, while the cellulose fiber (CLF) content was adjusted to 12wt% by using 12wt% fiber content composite pellets directly. The machine utilizes a pre-plunger system with a 10 mm diameter plunger and a mold clamping pressure of 29.4 kN. Table 2 presents the injection molding conditions, and Figure 4 displays the dimensions of the resulting specimens. The thickness of the molded product is 2 mm.

## 3. Methods

### 3.1. The IFSS Determination Method

**Figure 5:**
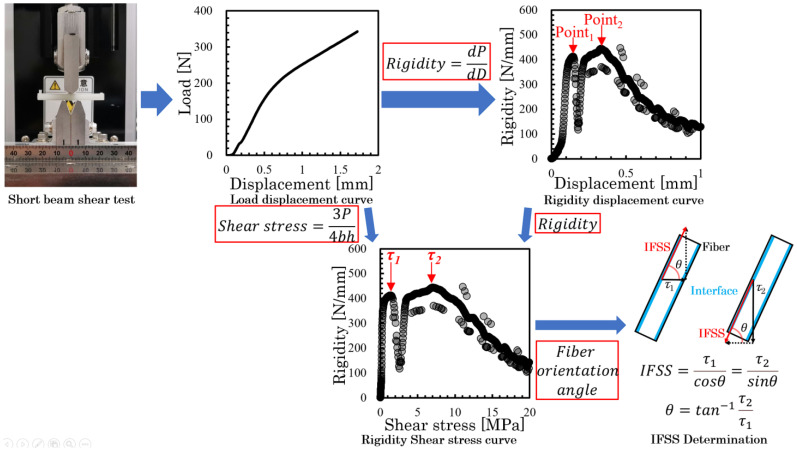
IFSS determination method flowchart.

Figure 5 presents the IFSS determination method-specific implementation process. The complete process from the load displacement curve obtained from the short beam shear test to the calculation of IFSS results was demonstrated. The specific theory will be explained in detail below. 

**Figure 6:**
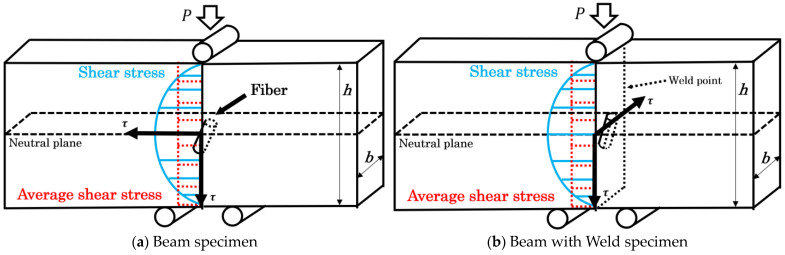
Deformation model of new methods for the Beam and Beam with Weld specimens.

**Figure 7:**
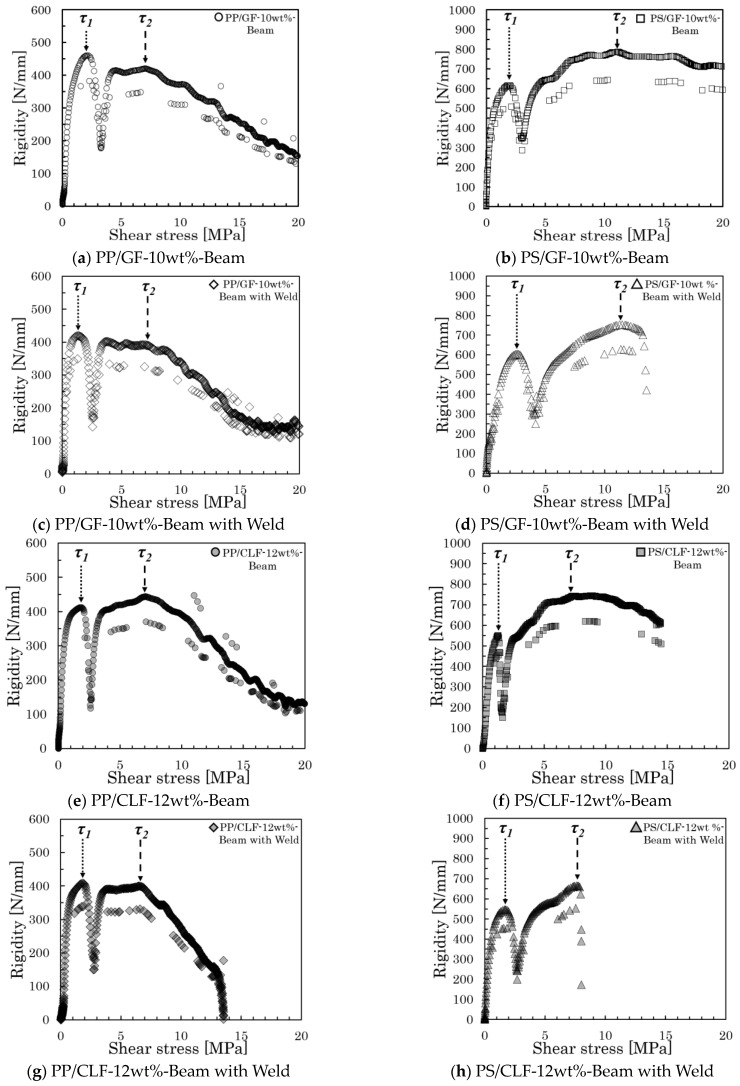
Rigidity–shear stress curves of specimens of all types.

The two types of specimens underwent a short beam shear test using a small universal mechanical testing machine (MCT-2150; A&D Co., Ltd., Tokyo, Japan) in accordance with ASTM D 2344M [30]. This test was conducted with a loading speed of 10 mm/min and a span of 10 mm. The specimen was placed edgewise in the direction of the support point. Figure 6 displays the shear stress diagram of the specimens. The shear stress, *τ*, was calculated using Equation (1):

(1) $$\tau =\frac{3P}{4bh}$$

where *P* denotes the load, *b* represents the specimen thickness, and *h* stands for the specimen’s width. The rigidity was obtained from the differential results of the load–displacement curve. To display the relationship between rigidity and shear stress more intuitively, we produced a rigidity–shear stress curve. Figure 7 presents all examples of the rigidity–shear stress curve of the specimens of two types.

**Figure 8:**
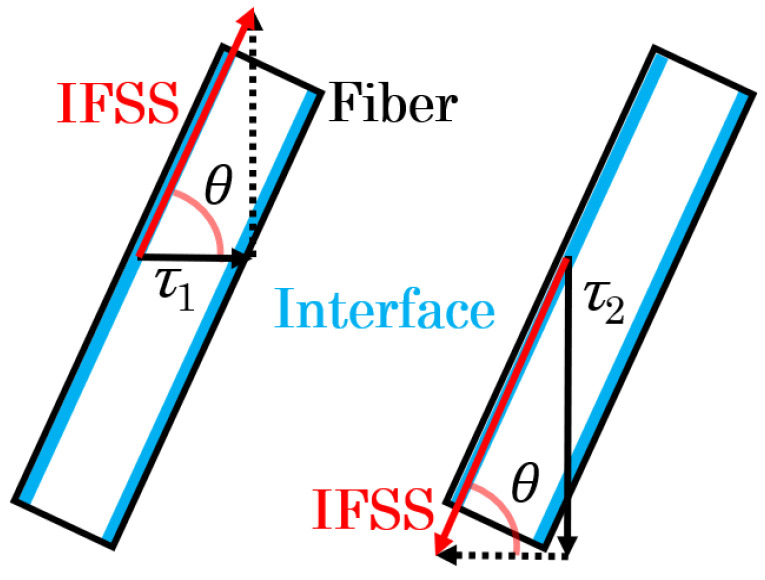
IFSS composite vector by average shear stress and fiber orientation angle.

However, when a three-point bending load is applied, a bending moment and shear stress are generated at the loading plane. The shear stresses are conjugate, reaching their maximum values on the neutral plane. In specimens of both types, high shear stress was generated near the neutral plane by reducing the spun length, which induces slippage at the interface. Furthermore, all rigidity–shear stress curves showed a discontinuous decrease in stiffness that was observed at two points. For obliquely oriented fibers, the interfacial slippage is regarded as occurring first in either the parallel or perpendicular direction to the loading direction; then, it arises in the opposite direction as the level of loading increases. Figure 8 presents the IFSS composite vector by the average shear stress and fiber orientation angle. Under the dotted line in that figure, a low level of shear stress is found where the rigidity reaches a discontinuous point—*τ*_1_; high shear stress is found where the rigidity reaches a discontinuous point—*τ*_2_. The relation between *τ*_1_, *τ*_2_, and *IFSS* is expressed by Equation (2):

(2) $$IFSS=\frac{\tau_{1}}{cos\theta }=\frac{\tau_{2}}{sin\theta }$$

where *θ* represents the fiber orientation angle of the GFs dispersed near the neutral plane. From Equation (2), *θ* is expressed by Equation (3).

(3) $$\theta =\tan^{-1}\frac{\tau_{2}}{\tau_{1}}$$

The *IFSS* can be obtained by substituting the *θ* obtained in Equation (3) into Equation (2). For this study, *IFSS* was calculated based on the method described above. The composition of each composite and different specimens’ type have been tested 10 times, and multiple sets of experiments were conducted to ensure the reproducibility of this experimental method.

### 3.2. Fiber Orientation Measurement

The fiber orientation corresponding to the core layer and weld point in the area subjected to the Beam and Beam with Weld specimens was photographed using a microfocus X-ray CT system (ScanXmate-D225RSS270; Comscantecno Co., Ltd., Yokohama, Japan). The obtained fiber orientation photographs were used to ascertain the average fiber orientation angle by choosing more than 250 fibers with respect to the core layer of the Beam specimens and the weld point of the Beam with Weld specimens using image analysis software (WinROOF ver.7.0.0; Mitani Corp., Fukui, Japan).

## 4. Results and Discussion

### 4.1. Comparison of IFSS Measurement Results of Two Types of Specimens

Table 3 presents the results of IFSS determination and earlier findings for these specimens, along with their percentages of deviation. The method section in this table indicates that Beam-2D and Beam with Weld were calculated using the IFSS calculation method outlined in Section 3.1. The results showed that the IFSS findings obtained from the specimens were similar to those obtained using the pull-out method and micro-droplet method and were slightly lower than the results obtained using the fragmentation method. The overall determination results fall within the same range. However, the standard deviation percentages for the pull-out, micro-droplet, and fragmentation methods were larger than 18%. The percentage deviation of the Beam specimens was approximately 9–17%, while that of the Beam with Weld specimens was less than 5%. Compared to earlier results obtained using other methods, the percentage deviation of the two types of specimens showed a marked decline. At the same time, we can also see that the previous IFSS evaluation results are different from the evaluation results of this study. Especially in the single fiber pull-out experiments, a higher level of IFSS was generally obtained, while in the micro-droplet experiments, a lower level of IFSS was generally obtained. This deviation can be attributed to changes in fiber volume fraction, and Jing Wang et al. also reached this conclusion by burying fibers of different lengths in the same volume of resin [15]. 

**Figure 9:**
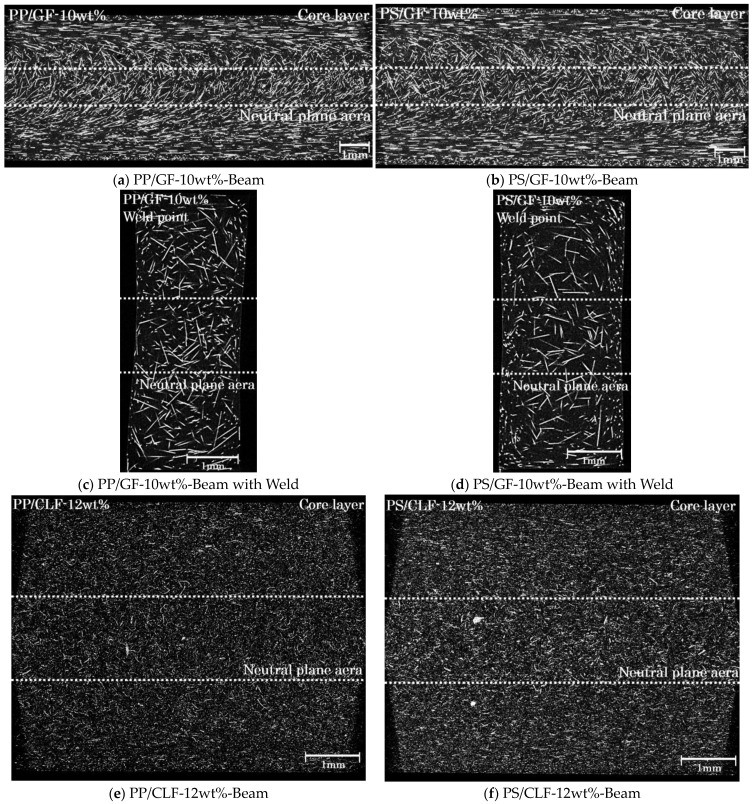

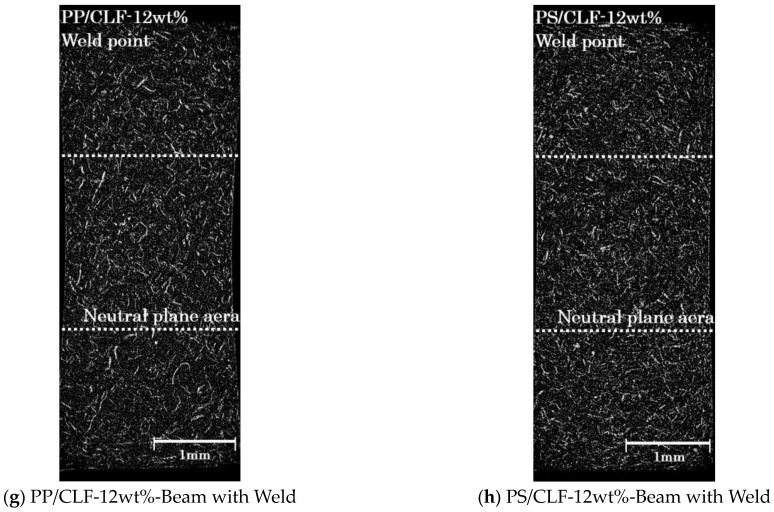
Fiber orientations of the Beam and Beam with Weld specimens.

For injection-molded single flow direction SFRTP products, the majority of fibers are oriented obliquely to the flow direction. Figure 9a,b show a GF orientation, while Figure 9e,f show a CLF orientation to the core layer near the neutral plane. The white part in the X-CT image is the fiber, and the black part is the matrix. Additionally, a weld was formed in the center by adjusting the flow path. At the weld point, the fibers are oriented vertically to the flow direction, resulting in a planar distribution of all fibers. Figure 9c,d present the orientation of a GF fiber, while Figure 9g,h present the orientation of a CLF near the neutral plane weld point.

**Figure 10:**
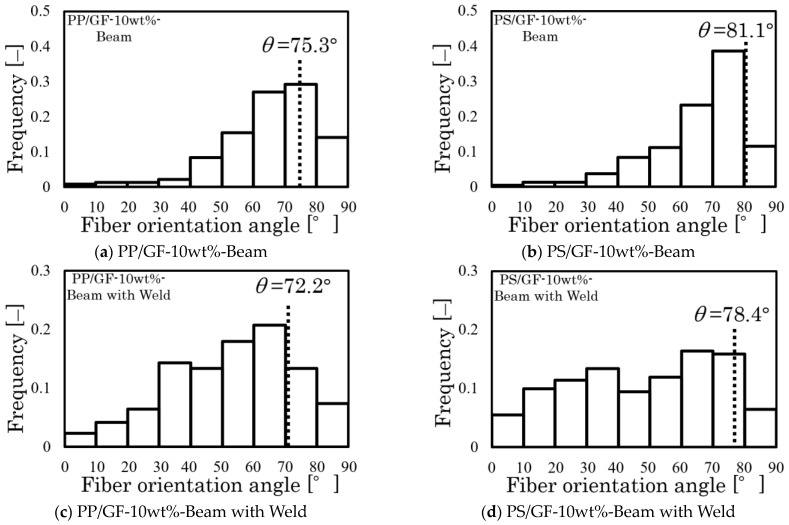

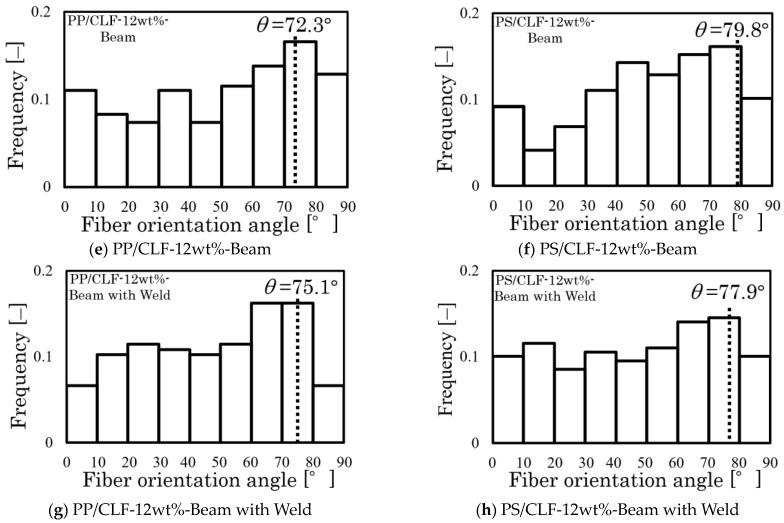
Fiber orientation angle distributions and fiber orientation angle results of the Beam and Beam with Weld specimens.

Figure 10 shows the distribution of fiber orientation angles measured from the X-ray CT photograph. The dotted line in this figure represents the fiber orientation angle results of the two types of specimens. The fiber orientation distribution and angle can be compared. The angle of fiber orientation approaches the azimuth near the high distribution frequency range. Additionally, the results are in complete agreement with those reported in the research conducted by the author and colleagues [26], indicating that this angle is a common orientation angle in these specimens.

**Figure 11:**
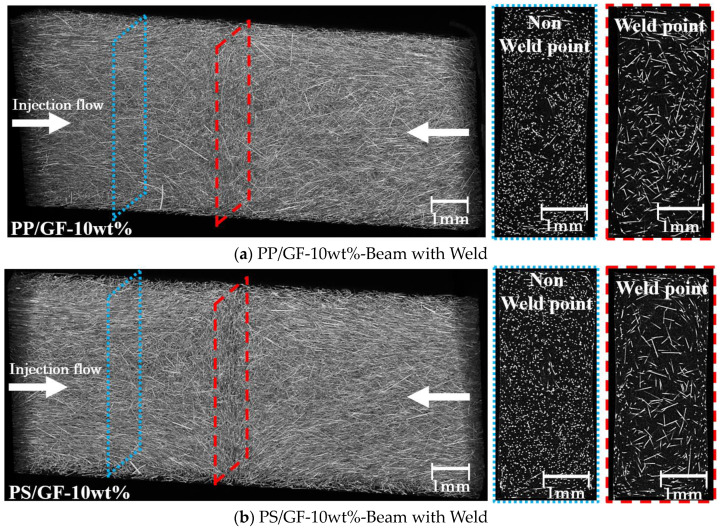

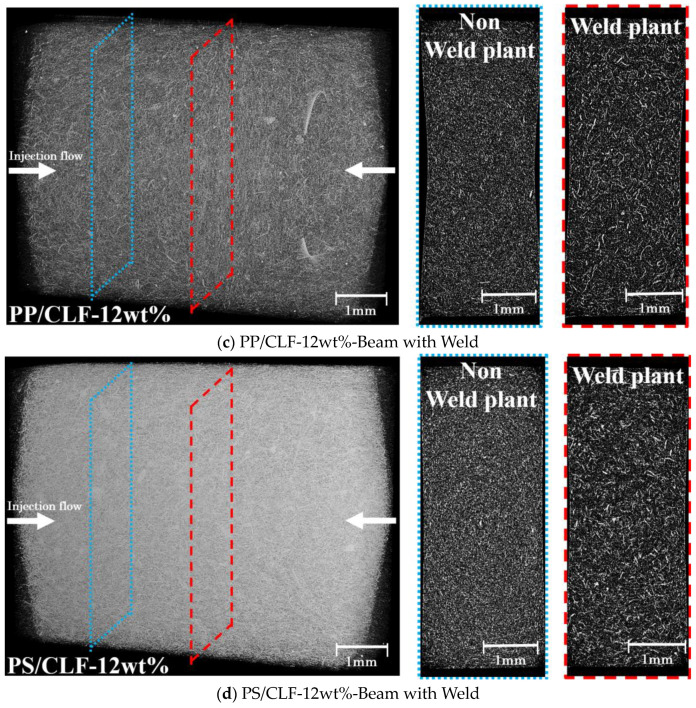
Three-dimensional Beam with Weld specimen fiber arrangement view and the weld point and non-weld point 2D image.

**Figure 12:**
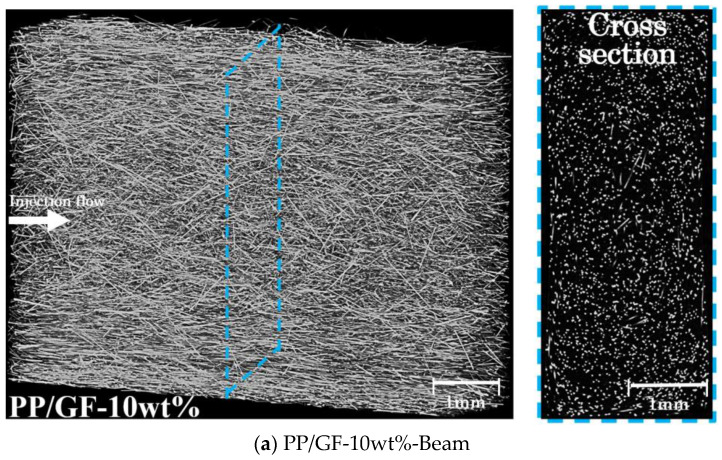

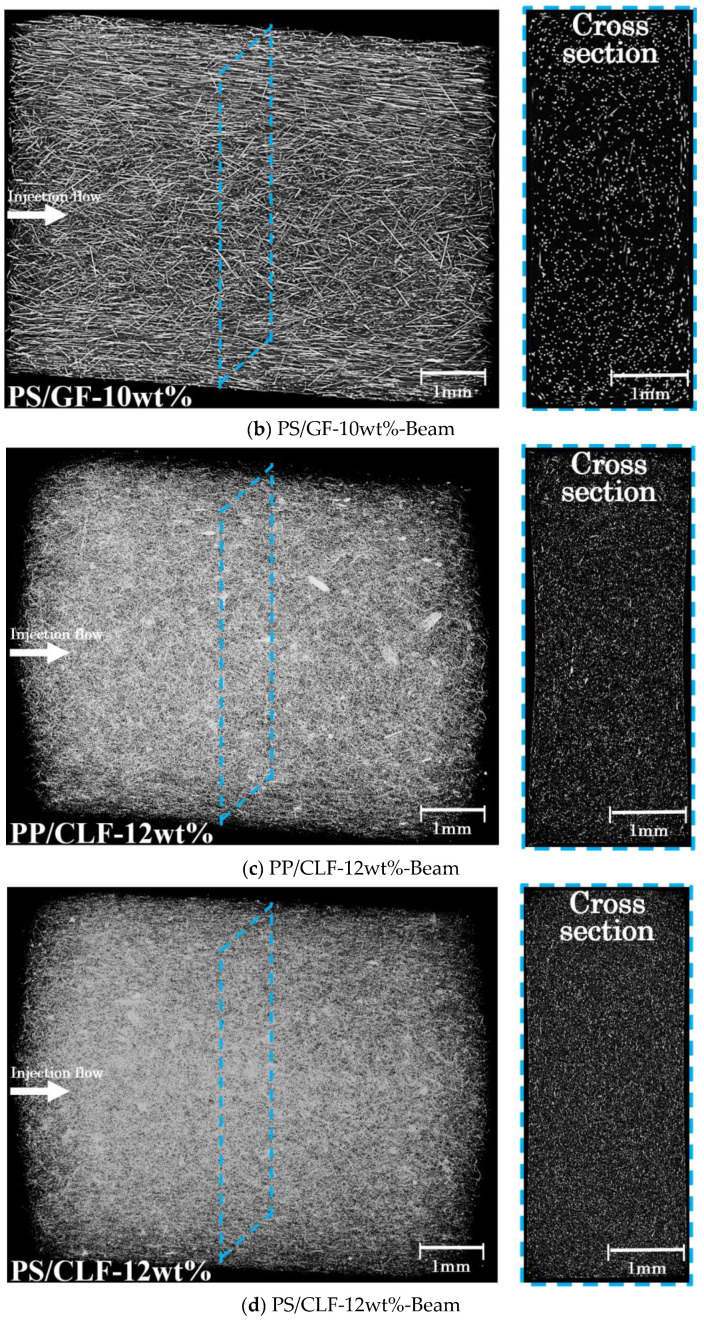
Three-dimensional Beam specimen fiber arrangement view and cross-sectional 2D image.

Combined with the previously described results, it is clear that the Beam with Weld specimens exhibit a lower percentage of deviation. This is primarily due to the weld point generated by adjusting the injection path, resulting in a two-dimensional fiber dispersion state. Figure 11 displays the three-dimensional view of the Beam with Weld specimen fiber arrangement, as well as the two-dimensional images of the weld point and non-weld point. In composite material compositions, fibers are dispersed in a two-dimensional plane at the weld point. Additionally, fibers in non-weld points are angled towards the direction of injection flow. For SFRTPs injection-molded products with a single flow direction, fibers in the molded products are randomly oriented. Figure 12 displays the 3D view of the beam specimen fiber arrangement and the 2D cross-sectional image of the injection-molded products of SFRTPs with a single flow direction. The 3D view and cross-sectional 2D image results show that the fibers in the core layer are dispersed in a triaxial-dimensional direction. Therefore, the IFSS determination may lead to a greater deviation. Based on these calculations and observations, the plane distribution of short fibers manufactured by welding joints can effectively reduce the range of measurement deviation. Compared to previous data, the interfacial shear strength of cellulose fibers is effectively reduced. Additionally, this text effectively avoids the influence of an increased deviation range of interfacial shear strength caused by factors such as uneven fiber radius and roughness of the fiber surface.

### 4.2. IFSS Triaxial Dimensional Orientation Angle Analytical Method

**Figure 13:**
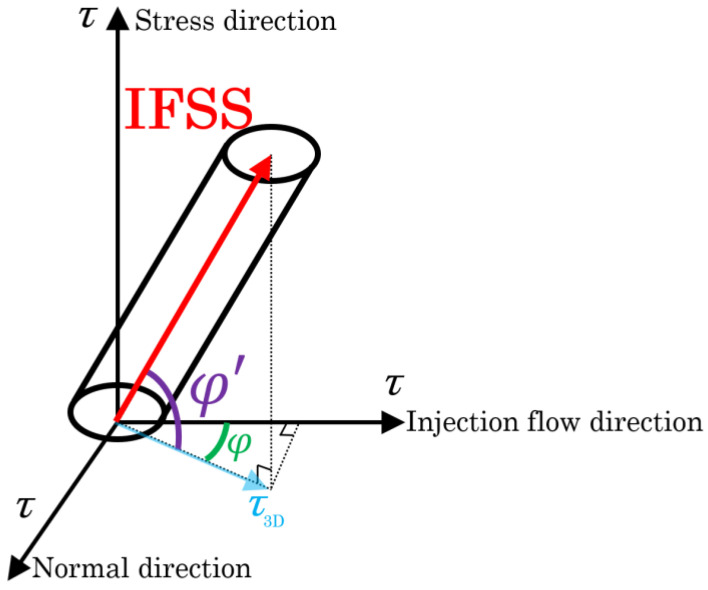
Beam specimen fiber shear stress diagram triaxial dimensional orientation.

**Figure 14:**
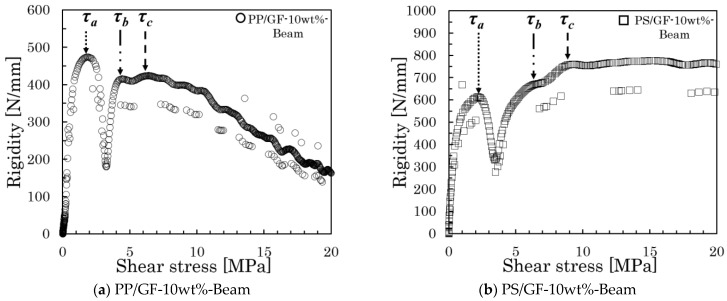

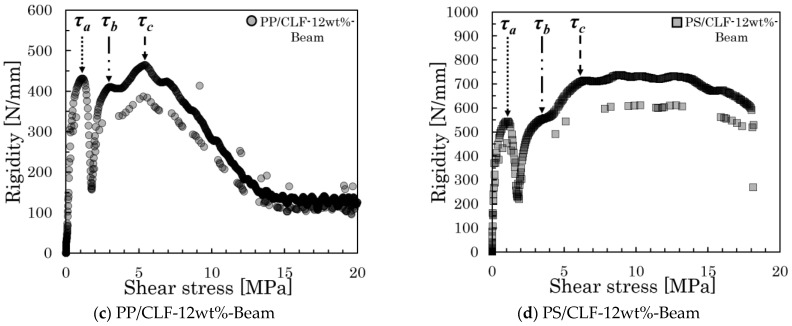
Triaxial-dimensional analysis rigidity-shear stress curves of Beam specimens.

In Section 4.1, we discussed the reliability of using Beam with Weld specimens to improve the accuracy of IFSS determination. The IFSS calculation results in Table 3 show that the calculation results of Beam-2D are slightly larger than those of Beam with Weld. The fiber exhibits a triaxial dimensional orientation in the unidirectional injection-molded short beam specimens [35]. The Beam-2D model, outlined in Section 4.1, does not consider the phenomenon of triaxial conjugation in the occurrence of shear stress. This numerical deviation can be explained by the observation in Figure 12 that the fiber has a triaxial dimensional fiber orientation. Figure 13 presents the IFSS composite vector by the average shear stress and fiber orientation angle of the Beam specimens in a triaxial dimensional orientation. As shown in this figure, the fiber is oriented triaxially within the neutral plane area of the injection-molded product. In fact, the rigidity–shear stress curve of the Beam specimens should exhibit three peak points. Figure 14 presents an example of the triaxial dimensional analysis rigidity–shear stress curves. These curves show three peak points of stress, from low to high. Combined with the stress analysis depicted in Figure 12, the first peak point can be called *τ_a_*, the second peak point *τ_b_*, and the projection of IFSS in the plane composed of the injection flow direction and normal direction as *τ*_3*D*_. *τ*_3*D*_ is expressed by Equation (4):

(4) $$\tau_{3D}=\frac{\tau_{a}}{sin\varphi }=\frac{\tau_{b}}{cos\varphi }$$

where *φ* represents the fiber orientation angle of the GF in the plane composed of the injection flow direction and normal direction. From Equation (4), *φ* is expressed by Equation (5) below.

(5) $$\varphi =\tan^{-1}\frac{\tau_{a}}{\tau_{b}}$$

**Figure 15:**
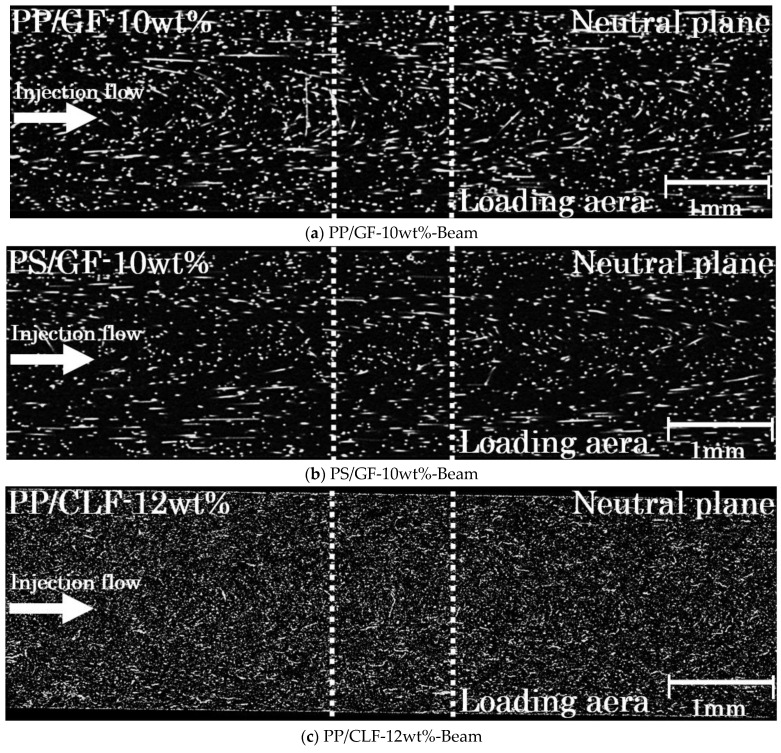

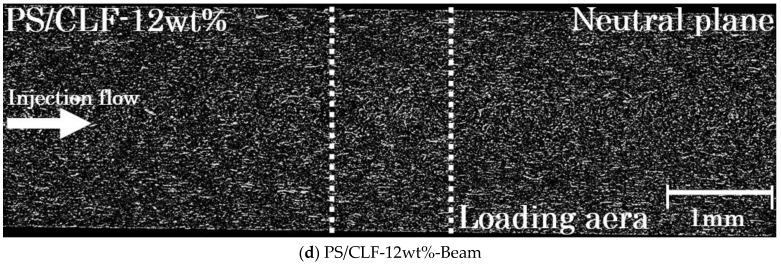
X-ray CT images of Beam specimens at the injection flow direction and the normal direction plane.

*τ*_3*D*_ can be calculated by substituting the obtained value of *φ* from Equation (5) into Equation (4). Similarly, the angle of fiber projection in the direction of stress can be calculated using the same method. Figure 15 shows the X-ray CT images of the Beam specimens at the injection flow direction and the normal direction plane. The white part in the X-CT image is the fiber, and the black part is the matrix. The line segment in Figure 15 represents the loading area of the Beam specimens in the neutral plane. The results showed that the fiber near the skin layer was almost parallel to the injection flow direction, while the fiber near the core layer presented a certain angle with the injection flow direction. The fiber orientation angle distribution was evaluated by selecting over 250 fibers in the loading area and measuring their frequency.

**Figure 16:**
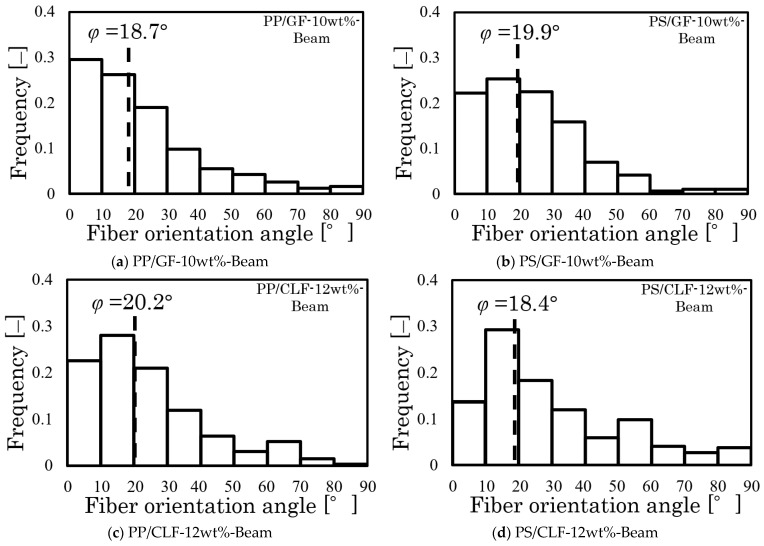
The injection flow direction and the normal direction plane fiber orientation angle distribution measured from the X-ray CT image.

Figure 16 displays the measured fiber orientation angle distribution from the X-ray CT image. This figure’s dotted line represents the fiber orientation angle results from Equation (5). The comparison of the fiber orientation distribution and the fiber orientation angle shows that the fiber orientation angle reaches the azimuth close to the high distribution frequency range. These results indicate that this *φ* represents a frequent orientation angle of the GF in the plane composed of the injection flow direction and normal direction. This implies that the *τ*_3*D*_ calculated by this angle can be used to calculate the triaxial dimensional IFSS. When combined with the stress analysis in Figure 12, the shear stress that reaches the third time peak rigidity for the curve can be defined as *τ_c_*. The relationship between *τ*_3*D*_, *τ_c_*, and IFSS is expressed by Equation (6):

(6) $$IFSS=\frac{\tau_{3D}}{{cos\varphi }^{\prime }}=\frac{\tau_{c}}{{sin\varphi }^{\prime }}$$

where *φ*′ represents the fiber orientation angle of the GF in the plane of the triaxial dimensional direction. From Equation (6), *φ*′ is expressed by Equation (7) below.

(7) $$\varphi^{\prime }=\tan^{-1}\frac{\tau_{c}}{\tau_{3D}}$$

The interfacial shear strength (IFSS) can be calculated by substituting the value of *φ*′ obtained in Equation (7) into Equation (6). Table 3 presents the results of IFSS determination and earlier findings for these specimens, along with their percentages of deviation. The method section in this table indicates that Beam-3D was calculated based on the triaxial dimensional direction method. The results showed that the IFSS findings obtained from the triaxial dimensional method are similar to those obtained using the pull-out method and micro-droplet method. The results of the Beam-3D test are more similar to those of Beam with Welds than Beam-2D, indicating that the triaxial dimensional direction of fibers should be considered when using Beam specimens, particularly for those using cellulose fibers. The deviation range was reduced by 7%. The IFSS calculation results of the PS/CLF-12wt% Beam specimens differ from the test results of the Beam with Weld specimens by only 0.4 MPa, indicating a significant improvement in accuracy and reliability. However, the calculation and curve value selection methods are relatively complex. For a simpler method of obtaining the IFSS of a fiber with greater stiffness, it is recommended to use the two-point method outlined in Section 3.1. The Beam-2D calculation method can obtain an IFSS with higher accuracy than the micro-droplet and pull-out methods, even without considering the triaxial stress state. For products with low fiber stiffness and easy bending during injection molding, it is recommended to use the Beam with Weld method for IFSS evaluation.

### 4.3. Imperfections of the Short Beam Shearing Method Using the Beam with Weld Specimens

**Figure 17:**
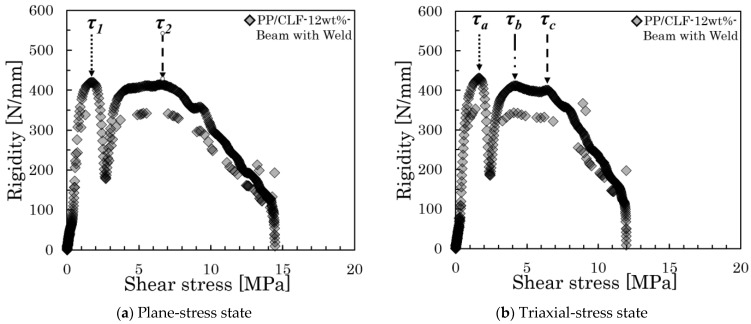
Different stress states in Beam with Weld specimens of PP/CLF-12wt%.

These results indicate that the short beam shear test using the Beam with Weld specimens in this study is highly precise and stable, making it suitable for application to both inorganic and organic fibers. However, the rigidity–shear stress curve obtained using the Beam with Weld specimens also reveals an obviously triaxial stress state. Figure 17 presents the rigidity–shear stress curve for two stress states that occurred in the PP/CLF-12wt% using the Beam with Weld specimens. The reasons for this result should be analyzed from two aspects: the test specimen itself and the test operation. Due to the random distribution of fibers in the melt during injection molding, the position of the weld point in all injection-molded products cannot be completely consistent. Additionally, the placement of the specimen in the short beam shear test is artificial, and visual judgments cannot ensure that the weld point is exactly in the position of the maximum average shear stress. The above reasons can lead to the maximum average shear stress occurring at a location away from the welding point during the actual loading process. In this study, eight out of ten testing results of Beam with Weld specimens can be classified as plane stress. These results indicate that while two stress states coexist, most of them belong to the plane stress state. This indicates that using Beam with Weld specimens is an effective method for controlling the plane stress state of fiber dispersion. In summary, it is necessary to analyze the fiber orientation state based on the shape of the stiffness–shear stress curve and apply different analytical methods for the 2D and 3D stress states.

## 5. Conclusions

This study proposes the use of a Beam with Weld test method to improve the calculation of interfacial shear strength (IFSS) by the short beam shear test. The results demonstrate that the short beam shear test using Beam with Weld specimens has high levels of precision and stability. Additionally, the IFSS calculation method of triaxial stress state analysis can also enhance the accuracy of calculation.

- It is noteworthy that IFSS can be directly calculated from injection-molded short fiber-reinforced thermoplastic polymers (SFRTPs).
- The results indicate that the IFSS determination findings from Beam-2D, Beam-3D, and Beam with Weld, as well as previous research results, fell within the same range.
- However, the percentage deviation (P.D.) from previous research results was larger than 18%, particularly when using cellulose fibers, where the P.D. was as high as 63%. On the other hand, when using the Beam-2D method, the P.D. was about 9–17%; when using the Beam-3D method, the P.D. was about 4–10%; and when using the Beam with Weld method, the P.D. was about 2–4%.
- The fiber orientation angle obtained represents the orientation angle of high-frequency fibers near the neutral plane of the core layer or loading area for Beam specimens and near the neutral plane of the weld point for Beam with Weld specimens.
- The analytical method for the rigidity–shear stress curve should depend on the fiber orientation state where the maximum average shear stress occurs during the test.
- Using the Beam with Weld method is an effective way to control the plane stress state of fiber dispersion.

This study has shown that the Beam with Weld method can improve the accuracy and stability of IFSS measurements and can effectively measure the interfacial shear strength of soft fibers, such as cellulose fibers. High-precision IFSS evaluations will provide an effective path for establishing quantitative relationships between mechanical properties such as tensile strength, bending strength, impact strength, and interfaces. The advantages of this method include, but are not limited to, controllable fiber volume fractions, applicability to multiple fibers or matrices, simple specimen fabrication, and simple experimental methods. This will further expand on the research related to interfacial strength. At the same time, we can anticipate the use of this method in short fiber-reinforced thermoplastics, including nanofibers, which are appropriate for injection molding. The focus of future research will be on the applicability of fibers and the impact of fiber volume fractions.

## Figures and Tables

**Table 1:** Mixing ratios under melt-mixing conditions.

Code	Mixing Temp. (°C)	PP (wt%)	PS (wt%)	GF (wt%)	CLF (wt%)
PP/GF-10wt%	230	90	-	10	-
PS/GF-10wt%		-	90	10	-
PP/CLF-12wt%	220	88	-	-	12
PS/CLF-12wt%		-	88	-	12

**Table 2:** Injection molding conditions.

Parameter	PP/GF-10wt%		PS/GF-10wt%		PP/CLF-12wt%		PS/CLF-12wt%	
Method	Beam	Beam with Weld	Beam	Beam with Weld	Beam	Beam with Weld	Beam	Beam with Weld
Injection temp. (°C)	230				200			
Mold temp. (°C)	50	70	50	70	50			
Injection speed (mm/s)	30	10	30	10	10			
Holding pressure (MPa)	84		92		84		92	
Injection time (s)	45		20		45		20	
Cooling time (s)	15							

**Table 3:** IFSS determination results obtained using a new method and earlier results.

Composite Material	Fiber Content (wt%)	Method	IFSS (MPa)	Deviation (%)	Source
PP/GF	10wt%	Beam-2D	7.4 ± 0.7	9	This work
	10wt%	Beam-3D	7.3 ± 0.4	5	This work
	10wt%	Beam with Weld	7.3 ± 0.2	3	This work
	Single fiber	Pull-out	6.8 ± 1.7	25	[9]
	7wt%	Push-out	11.8 ± 2.4	20	[10]
	Single fiber	Fragmentation	9.5 ± 1.9	20	[11]
	Single fiber	Micro-droplet	7.2 ± 1.3	18	[9]
PS/GF	10wt%	Beam-2D	11.2 ± 1.1	10	This work
	10wt%	Beam-3D	11.2 ± 0.4	4	This work
	10wt%	Beam with Weld	11.0 ± 0.2	2	This work
	Single fiber	Pull-out	12.8 ± 2.3	18	[10]
	Single fiber	Micro-droplet	11.7 ± 2.3	20	[9]
PP/CLF	12wt%	Beam-2D	7.1 ± 1.0	14	This work
	12wt%	Beam-3D	7.0 ± 0.5	7	This work
	12wt%	Beam with Weld	6.9 ± 0.3	4	This work
	Single fiber	Fragmentation	6.3 ± 4.0	63	[31]
	Single fiber	Pull-out	9.1 ± 4.7	51	[31]
	Single fiber	Micro-droplet	6.1 ± 2.2	36	[32]
PS/CLF	12wt%	Beam-2D	8.2 ± 1.4	17	This work
	12wt%	Beam-3D	7.8 ± 0.8	10	This work
	12wt%	Beam with Weld	7.6 ± 0.3	4	This work
	Single fiber	Micro-droplet	7.0 ± 2.0	29	[33]
	Single fiber	Fragmentation	10.2 ± 2.2	22	[34]

## Data Availability

Data are contained within the article.
